# Supplementary material for: Lived experiences of postpartum hypertensive women in Accra, Ghana
Source: PLoS One. 2026 May 27;21(5):e0349781. doi: 10.1371/journal.pone.0349781 (PMC13215485; doi:10.1371/journal.pone.0349781)
Supplement: S1 File — (DOCX) [file pone.0349781.s001.docx]

Other

# **Appendix 1: INTERVIEW QUESTIONS**

**SECTION A: Demographic and Background Information**

1. Can you tell me a bit about yourself (e.g., age, marital status, number of children)?
2. How long ago did you deliver?
3. When were you diagnosed with hypertension? Within or after pregnancy.
4. Describe the treatment you are currently receiving for your hypertension.

**SECTON B: Expectations**

1. Describe the advantages you expect from managing your hypertension well.
2. In your view, describe what can happen if the hypertension is not managed properly?
3. Has your experience with managing hypertension met your expectations so far?

How

**SECTION C: Self-Efficacy in Hypertension Management**

1. How do you feel about your ability to manage your hypertension after giving birth?
2. Describe the kinds of things you do to take care of your condition.
3. Describe the challenges you face in the management of your condition.

**SECTION D: Environmental Influences.**

11. How does your family support the management of your hypertension?

12. How easy is it to access healthcare or medications for your condition?
 13. Describe the support do you receive from health care providers?

**SECTION E: Observational Learning and Coping Strategies**

14. Describe what you have learnt from others in the management of hypertension?

15. Can you describe any person or experience that influenced how you currently manage your

condition?

16. Describe what you would want other postpartum women with hypertension to know about

coping and managing it?

**SECTION F: Reflection and Coping**

16. How has living with the condition affected you emotionally, physically, socially, or spiritually

since childbirth?

18. Describe what helps you cope within difficult moments?

19. Is there anything else you would like to share regarding your experience in the management

of your condition?

Thank you.
