# Supplementary material for: Lived experiences of postpartum hypertensive women in Accra, Ghana
Source: PLoS One. 2026 May 27;21(5):e0349781. doi: 10.1371/journal.pone.0349781 (PMC13215485; doi:10.1371/journal.pone.0349781)
Supplement: Attachment — Submitted filename: PONE-D-25-62035_reviewer comments.pdf [file pone.0349781.s002.pdf]

PLOS ONE

Lived experiences of postpartum women diagnosed with hypertension receiving care in a resource-constrained setting in Accra, Ghana

--Manuscript Draft--

**Manuscript Number:**	PONE-D-25-62035
**Article Type:**	Research Article
**Full Title:**	Lived experiences of postpartum women diagnosed with hypertension receiving care in a resource-constrained setting in Accra, Ghana
**Short Title:**	Lived experiences of postpartum women diagnosed with hypertension receiving care in Accra
**Corresponding Author:**	Kennedy Dodam Konlan, PhD<br>University of Ghana<br>Accra, Greater Accra GHANA
**Keywords:**	Hypertension, Ghana, Lived-Experiences, Post-Partum, Self-efficacy
**Abstract:**	Background: Hypertension is a leading cause of adult mortality globally, with postpartum hypertension posing distinct risk to women and yet it is a frequently neglected challenge. In Ghana, particularly within the Accra Metropolis, there is a limited understanding of the lived experiences of women managing hypertension after childbirth<br><br>Aim: We described the lived experiences of postpartum women diagnosed with hypertension receiving care in a resource-constrained setting in Accra, Ghana<br><br>Methods: The study employed a qualitative, descriptive- phenomenological design. We purposive selected 16 postpartum women diagnosed with hypertension and receiving care recruited from the postnatal clinic of the Greater Accra Regional Hospital. The data was collected via in-depth, semi-structured interviews using pretested interview guide. The interviews were audio-taped and transcribed verbatim. We then conducted thematic analysis with the aid NVivo 11.0.<br><br>Results: The analysis yielded three major themes; expectations about living with hypertension, self-efficacy in managing hypertension and environmental factors on the management of hypertension. On the expectation about living with hypertension, we identified the following sub-themes; optimism and hope for recovery and fear and uncertainty about complications. Regarding self-efficacy, the participants demonstrated varying degrees of confidence in managing their condition and the following sub-themes were identified: Confidence in adhering to treatment and challenges with lifestyle modification and monitoring. On the environmental factors influencing management of hypertension among the participants, we found the following sub-themes, spousal and family support, role of healthcare providers, social perceptions and stigma.<br><br>Conclusion and recommendations: Effective management of postpartum hypertension requires patient-centered and holistic interventions that enhance women's self-efficacy, strengthen family and community support, reduces stigma and improve midwife–client communication. It is recommended that the Ghana Health Service design targeted postpartum hypertension education programs aimed at reducing social stigma and integrates the role of spouses and social networks of clients including faith-based organizations to improve compliance with treatment and lifestyle modifications.
**Order of Authors:**	Kennedy Dodam Konlan, PhD
	Cecilia Eliason, PhD
	Hellen Akosua Asante, Mphil Midwifery
**Additional Information:**	
**Question**	**Response**

Powered by Editorial Manager® and ProduXion Manager® from Aries Systems Corporation

**Financial Disclosure**<br><br>Enter a financial disclosure statement that describes the sources of funding for the work included in this submission. Review the submission guidelines for detailed requirements. View published research articles from *PLOS ONE* for specific examples.<br><br>This statement is required for submission and **will appear in the published article** if the submission is accepted. Please make sure it is accurate.<br><br>**Funded studies**<br>Enter a statement with the following details:<br>• Initials of the authors who received each award<br>• Grant numbers awarded to each author<br>• The full name of each funder<br>• URL of each funder website<br>• Did the sponsors or funders play any role in the study design, data collection and analysis, decision to publish, or preparation of the manuscript?<br><br>Did you receive funding for this work?	The author(s) received no specific funding for this work.
**Competing Interests**<br><br>Use the instructions below to enter a competing interest statement for this submission. On behalf of all authors, disclose any competing interests that could be perceived to bias this work—acknowledging all financial support and any other relevant financial or non-financial competing interests.<br><br>This statement is required for submission and **will appear in the published article** if the submission is accepted. Please make sure it is accurate and that any funding sources listed in your Funding Information later in the submission form are also declared in your Financial Disclosure statement.	The authors have declared that no competing interests exist.

View published research articles from *PLOS ONE* for specific examples.

NO authors have competing interests

Enter: *The authors have declared that no competing interests exist.*

Authors with competing interests

Enter competing interest details beginning with this statement:

I have read the journal's policy and the authors of this manuscript have the following competing interests: [insert competing interests here]

* typeset

Ethics Statement

Enter an ethics statement for this submission. This statement is required if the study involved:

- Human participants
- Human specimens or tissue
- Vertebrate animals or cephalopods
- Vertebrate embryos or tissues
- Field research

Write "N/A" if the submission does not require an ethics statement.

General guidance is provided below. Consult the submission guidelines for detailed instructions. **Make sure that all information entered here is included in the Methods section of the manuscript.**

Ethical clearance was obtained from the Ghana Health Service Ethics Review Committee
(GHS – ERC:028/ 06/ 2025), supported by a letter from the School of Nursing, University of
Ghana. Potential participants were given information sheets detailing the purpose, objectives,
possible benefits, risks, and inconveniences associated with the study. Confidentiality, voluntary participation, and the right to withdraw at any point were emphasized. Interviews
were conducted in English at venues preferred by participants. A follow-up was made to
confirm participation. Informed consent forms were signed by participants who agreed to take
part. For those unable to read or write, forms were translated into their preferred local language
in the presence of a witness, after which both the participant and witness signed or thumbprinted
the form. Participants were informed that even after signing, they retained the right to withdraw without repercussions. Anonymity was guaranteed by assigning codes to participants
in the order of recruitment, which were later replaced with pseudonyms in the findings section.
Collected information, including consent forms and recordings, will be securely stored for at
least five years after the study. Demographic data will be kept separately from interview
records. Electronic files will be stored in password-protected folders. In cases where participants became emotionally distressed while recounting their experiences, arrangements
were made for professional counseling support.

Powered by Editorial Manager® and ProduXion Manager® from Aries Systems Corporation

**Format for specific study types**<br><br>**Human Subject Research (involving human participants and/or tissue)**<br>• Give the name of the institutional review board or ethics committee that approved the study<br>• Include the approval number and/or a statement indicating approval of this research<br>• Indicate the form of consent obtained (written/oral) or the reason that consent was not obtained (e.g. the data were analyzed anonymously)<br><br>**Animal Research (involving vertebrate animals, embryos or tissues)**<br>• Provide the name of the Institutional Animal Care and Use Committee (IACUC) or other relevant ethics board that reviewed the study protocol, and indicate whether they approved this research or granted a formal waiver of ethical approval<br>• Include an approval number if one was obtained<br>• If the study involved *non-human primates*, add *additional details* about animal welfare and steps taken to ameliorate suffering<br>• If anesthesia, euthanasia, or any kind of animal sacrifice is part of the study, include briefly which substances and/or methods were applied<br><br>**Field Research**<br><br>Include the following details if this study involves the collection of plant, animal, or other materials from a natural setting:<br>• Field permit number<br>• Name of the institution or relevant body that granted permission	
**Data Availability**<br><br>Authors are required to make all data underlying the findings described fully available, without restriction, and from the time of publication. PLOS allows rare exceptions to address legal and ethical concerns. See the PLOS Data Policy and FAQ for detailed information.	Yes - all data are fully available without restriction

Powered by Editorial Manager® and ProduXion Manager® from Aries Systems Corporation

A Data Availability Statement describing where the data can be found is required at submission. Your answers to this question constitute the Data Availability Statement and **will be published in the article**, if accepted.<br><br>**Important:** Stating 'data available on request from the author' is not sufficient. If your data are only available upon request, select 'No' for the first question and explain your exceptional situation in the text box.<br><br>Do the authors confirm that all data underlying the findings described in their manuscript are fully available without restriction?	
**Describe where the data may be found in full sentences. If you are copying our sample text, replace any instances of XXX with the appropriate details.**<br><br>• If the data are **held or will be held in a public repository**, include URLs, accession numbers or DOIs. If this information will only be available after acceptance, indicate this by ticking the box below. For example: *All XXX files are available from the XXX database (accession number(s) XXX, XXX.).*<br>• If the data are all contained **within the manuscript and/or Supporting Information files**, enter the following: *All relevant data are within the manuscript and its Supporting Information files.*<br>• If neither of these applies but you are able to provide **details of access elsewhere**, with or without limitations, please do so. For example:<br><br>*Data cannot be shared publicly because of [XXX]. Data are available from the XXX Institutional Data Access / Ethics Committee (contact via XXX) for researchers who meet the criteria for access to confidential data.*<br><br>*The data underlying the results presented in the study are available from (include the name of the third party*	All relevant data are within the manuscript and its Supporting Information files.

and contact information or URL).<br>• This text is appropriate if the data are owned by a third party and authors do not have permission to share the data.<br><br>* typeset	
Additional data availability information:	

Manuscript
Click here to access/download;Manuscript;Manuscript clean file.docx

Lived experiences of postpartum women diagnosed with hypertension receiving care in a resource-constrained setting in Accra, Ghana

Hellen Akosua Asante [1], Cecilia Eliason[1], Kennedy Dodam Konlan [1]

1. Department of Adult Health, School of Nursing and Midwifery, University of Ghana, Legon, Greater Accra Region, Ghana

Corresponding Author: Dr. Kennedy Dodam Konlan, Department of Adult Health, School of Nursing and Midwifery, University of Ghana, Legon, Greater Accra Region, Ghana. Email: kennedy.konlan@gmail.com

Author's email

Hellen Akosua Asante- hellenakosua15@gmail.com , Dr. Cecilia Eliason- celiason@ug.edu.gh

ABSTRACT

Background: Hypertension is a leading cause of adult mortality globally, with postpartum hypertension posing distinct risk to women and yet it is a frequently neglected challenge. In Ghana, particularly within the Accra Metropolis, there is a limited understanding of the lived experiences of women managing hypertension after childbirth

Aim: We described the lived experiences of postpartum women diagnosed with hypertension receiving care in a resource-constrained setting in Accra, Ghana

Methods: The study employed a qualitative, descriptive- phenomenological design. We purposive selected 16 postpartum women diagnosed with hypertension and receiving care recruited from the postnatal clinic of the Greater Accra Regional Hospital. The data was collected via in-depth, semi-structured interviews using pretested interview guide. The interviews were audio-taped and transcribed verbatim. We then conducted thematic analysis with the aid NVivo 11.0.

Results: The analysis yielded three major themes; expectations about living with hypertension, self-efficacy in managing hypertension and environmental factors on the management of hypertension. On the expectation about living with hypertension, we identified the following sub-themes; optimism and hope for recovery and fear and uncertainty about complications. Regarding self-efficacy, the participants demonstrated varying degrees of confidence in managing their condition and the following sub-themes were identified: Confidence in adhering to treatment and challenges with lifestyle modification and monitoring. On the environmental factors influencing management of hypertension among the participants, we found the following sub-themes, spousal and family support, role of healthcare providers, social perceptions and stigma.

Conclusion and recommendations: Effective management of postpartum hypertension requires patient-centered and holistic interventions that enhance women's self-efficacy, strengthen family and community support, reduces stigma and improve midwife–client communication. It is recommended that the Ghana Health Service design targeted postpartum hypertension education programs aimed at reducing social stigma and integrates the role of spouses and social networks of clients including faith-based organizations to improve compliance with treatment and lifestyle modifications.

Key words: Hypertension, Ghana, Lived-Experiences, Post-Partum, Self-efficacy

Introduction

Hypertensive disorders remain among the foremost causes of maternal morbidity and mortality globally [1-7], yet the postpartum period continues to receive limited attention despite its high-risk nature [8]. Postpartum hypertension, which may represent persistent or new-onset high blood pressure following childbirth, is increasingly recognized as a critical maternal health issue [2, 8]. Recent studies [1-8] estimate that approximately 28.4% of women who experience hypertensive disorders of pregnancy continue to have hypertension within two years postpartum, compared with 9.1% of those without prior hypertensive disorders [9]. In low-resource settings, as many as 62.1% of women with preeclampsia or eclampsia remain hypertensive one year after delivery [1,10]. Moreover, even among women with uncomplicated pregnancies, about 3% develop de novo hypertension within the first postpartum year [8, 11].

In Africa, the burden of postpartum hypertension is notably higher due to delayed diagnosis, limited access to quality prenatal and postnatal care, and inadequate treatment strategies [12-14]. For instance, research in Nigeria revealed that 12% of postpartum women developed hypertension, a situation worsened by weak health systems and cultural influences [14]. In Uganda, prevalence was reported at 14%, largely attributed to gaps in follow-up care [14] while in South Africa, around 10% of postpartum women were affected, with striking differences in healthcare access between rural and urban areas [8, 14].In Ghana, available data points to a rising trend, with estimates suggesting 10–15% of mothers experience postpartum hypertension, particularly in urban centers such as Accra [15]. Similar figures were found in other African countries: 11% in Kenya, where inadequate monitoring was a key contributor [14], and 13% in Ethiopia, where late diagnosis often led to life-threatening complications [8, 16].

In Ghana, hypertensive disorders in pregnancy continue to impose a significant burden on maternal health. A recent study reported a prevalence rate of 37.2% among antenatal clinic attendees, with 17.6% presenting chronic hypertension superimposed on preeclampsia [17]. These statistics underscore the need for continued postpartum follow-up, as unresolved or newly developed hypertension after delivery contributes to long-term cardiovascular, renal, and cerebrovascular complications [9, 18]. Beyond its physical health implications, postpartum hypertension also exerts considerable psychological, social, and economic pressures on mothers, particularly in resource-constrained contexts. Many affected women struggle to adhere to antihypertensive medications while balancing newborn care, follow-up

appointments, and limited social or family support [11, 19]. The compounded stress and anxiety may further compromise health outcomes and family stability [18-20]. Despite growing recognition of postpartum hypertension as a major maternal health concern, limited qualitative research have explored the lived experiences of postpartum women diagnosed with hypertension and receiving care in Ghana. This gap in understanding hampers the ability of healthcare professionals and policymakers to design holistic, woman-centered interventions that address both the clinical and psychosocial needs of postpartum women living with hypertension in resource-constrained settings like those in Ghana.

Aim

We described the lived experiences of postpartum women diagnosed with hypertension receiving care in a resource-constrained setting in Accra, Ghana

Method

Study design

The study employed a descriptive phenomenological approach to investigate the lived experiences of postpartum women diagnosed with hypertension in the Accra Metropolis. Grounded in Edmund Husserl's philosophical perspective, descriptive phenomenology seeks to uncover the essence of human experiences by suspending researcher assumptions through

Study setting

The study was conducted at the Greater Accra Regional Hospital (GARH), commonly referred to as Ridge Hospital, situated at No. 1 Haile Selassie Street, North Ridge, Accra. The facility is centrally located, making it accessible from different parts of the city. The Greater Accra Region has a population of about 5,055,883, with females representing 50.7% of the national population [5, 6]. It is one of the most densely populated regions, with an estimated 1,205 people per square kilometer. The region has an extensive network of public and private health facilities. GARH was selected as the study site because of its strategic role as a major referral center in the region and its high caseload of pregnant and postpartum women, particularly those with hypertensive disorders [18, 20]. Data suggest that approximately 8.5% of pregnant women attending the facility experience hypertensive conditions [19]. This setting provided access to a diverse pool of participants and offered an opportunity to examine the phenomenon within a busy clinical environment.

Target population and sample size determination

The study population consisted of all postpartum women diagnosed with hypertension who were receiving postnatal care at GARH.

The sample size was determined at data saturation which was achieved at the conclusion of the sixteenth (16th) interview. We observed data redundancy after the conclusion of the twelveth interview and after concluding four additional interviews, we observed that no new information was being elicited hence data saturation at the 16th interview.

Inclusion criteria:

- Women with a confirmed diagnosis of hypertension (≥140/90 mmHg) on at least three separate measurements while at rest.
- Attending postnatal clinic at GARH.
- Cognitively stable.

Exclusion criteria:

- Women too ill to participate.
- Those with hearing or speech impairments preventing participation.

Selection of participants and data collection

Participants were selected if they were diagnosed with hypertension during the postpartum period. This approach enabled us to identify individuals who could offer valuable insights into their experiences living with postpartum hypertension, thereby enriching the qualitative data for the study.

The data collection took place between 4th October 2025 to 30th October 2025**.** Ethical approval was obtained from the Ghana Health Service Ethics Review Committee (GHS – ERC: 028/06/25). An introductory letter from the School of Nursing, University of Ghana, was presented to the hospital to facilitate access. Nurses at the postnatal clinic were briefed on the study’s objectives and eligibility criteria. Potential participants were identified by the the researcher team, who then contacted them, explained the study in detail, answered their questions, and screened them for eligibility. Interested women were given information sheets

and consent forms. Appointments were arranged at convenient times and locations for interviews. All the participants agreed to grant the interviews at a consulting room assigned to the searchers at the postnatal clinic.

The participants were interviewed using a semi-structured interview guide (S2). The interview guide consisted of two sections: the first captured demographic information, while the second focused on the participants' expectations and lived experiences related to living with postpartum hypertension. Each interview began with a broad, introductory ("grand tour") question to encourage open discussion, followed by targeted probes to explore specific areas of experience in greater depth. Field notes were also taken to capture non-verbal cues and contextual observations.

The interviews lasted between 30-45 minutes and were conducted in English (the official language of communication in Ghana) and two native Ghanaian languages widely spoken in Accra (Twi and Ga). The interviews were recorded using an audio recorder, transcribed verbatim after each interview. We further did member checking with the participants to ensure their exact views had been captured.

Data Management

To maintain confidentiality, pseudonyms were assigned to participants (e.g., names of the alphabets were used). Audio recordings were transcribed verbatim immediately after interviews. Files were stored with unique identifiers, and transcripts were separated from demographic details. Hard copies were kept securely, while digital files were password-protected on the researcher's computer.

Methodological Rigor

To strengthen the accuracy and dependability of the results, the researcher implemented measures consistent with the four recognized principles of trustworthiness, credibility, dependability, confirmability, and transferability as outlined in contemporary qualitative research standards.

Credibility was strengthened through prolonged engagement, member checking, and triangulation. Prolonged engagement involved building trust with mothers during clinic visits before interviews were conducted. Member checking allowed participants to confirm or clarify

interpretations of their responses, ensuring accuracy of representation. In addition, peer debriefing with fellow researchers and supervisors was carried out to refine emerging themes, which increased the authenticity of findings.

Dependability was ensured through the creation of a detailed audit trail that documented each step of the research process, including interview procedures, field notes, transcription records, and analytical decisions. This systematic documentation provides transparency and allows other researchers to understand how the findings were derived. The researcher also maintained a reflective journal throughout the study to record decisions, challenges, and insights that emerged during data collection and analysis. This practice contributed to consistency and accountability in the research process.

Confirmability, which focuses on minimizing researcher bias and ensuring that findings are grounded in participants' perspectives rather than the researcher's assumptions, was achieved through reflexivity. The researcher acknowledged her professional background as a midwife and consciously bracketed personal experiences and preconceived ideas about postpartum hypertension. Regular self-reflection and adherence to the interview guide helped maintain neutrality during data collection and analysis. Furthermore, the inclusion of verbatim quotes from participants in the findings ensured that interpretations were rooted in their authentic voices rather than subjective inference.

Transferability was promoted through detailed descriptions of the study context, participants' demographic details, and factors influencing postpartum hypertension. Although statistical generalization is not the aim of qualitative work, providing rich contextual detail allows readers to judge the applicability of findings to similar settings.

Finally, ethical rigour was ensured through informed consent, confidentiality, and securing ethical approval from the Ghana Health Service Ethics Review Committee, supported by an introductory letter from the School of Nursing, University of Ghana. Participants were reminded of their right to withdraw from study at any stage without any consequences. Collectively, these measures enhanced the trustworthiness of the inquiry.

Ethical approval and consent to participate.

Ethical clearance was obtained from the Ghana Health Service Ethics Review Committee (GHS – ERC:028/ 06/ 2025), supported by a letter from the School of Nursing, University of Ghana. Potential participants were given information sheets detailing the purpose, objectives, possible benefits, risks, and inconveniences associated with the study. Confidentiality, voluntary participation, and the right to withdraw at any point were emphasized. Interviews were conducted in English at venues preferred by participants. A follow-up was done to confirm participation. Informed consent forms were signed by participants who agreed to take part. For those unable to read or write, forms were translated into their preferred local language in the presence of a witness, after which both the participant and witness signed or thumb-printed the form. Participants were informed that even after signing, they retained the right to withdraw without repercussions. Anonymity was guaranteed by assigning codes to participants in the order of recruitment, which were later replaced with pseudonyms in the findings section. Collected information, including consent forms and recordings, is securely stored for at least five years after the study. Demographic data will be kept separately from interview records. Electronic files will be stored in password-protected folders. In cases where participants became emotionally distressed while recounting their experiences, arrangements were made for professional counselling support.

Data Analysis

In this study, analysis was conducted alongside data collection. Each interview was analyzed before the next was conducted, allowing emerging insights to inform subsequent interviews and enhance the richness of the data. All interviews were audio-recorded and listened to several times to ensure familiarity and immersion in the data. Interviews conducted in Twi and Ga were translated and transcribed into English with the assistance of qualified language experts in Twi and Ga, ensuring accuracy, tone, and cultural meaning were maintained. The translated transcripts were compared with the original Twi and Ga recordings to confirm that participants' intentions and emotions were preserved. The transcribed data were analyzed using the descriptive phenomenological method, which involves reading the transcripts to gain an overall sense of the data, identifying significant statements, transforming these statements into meaningful units, grouping similar meanings into clusters, and organizing them into overarching themes that capture the essence of participants' experiences. NVivo version 11 software was used to systematically manage and organize the codes to enhance transparency

and rigor. Themes were generated around the key areas of expectations, self-efficacy and environmental factors based on the social cognitive theoretical framework.

Results

The study involved sixteen postpartum mothers aged between 21 and 42 years, reflecting a broad range of reproductive experiences. In terms of parity, the majority had between two and four children, while a few were first-time mothers. At the time of the interviews, participants were between one week and seven months postpartum, representing varying stages of recovery and adjustment after childbirth. Most women shared that their hypertension was first detected during pregnancy, whereas a few reported its onset after delivery. Their management primarily involved Neficard (XL 30–90 mg) and Methyldopa, either taken singly or in combination, under the guidance of healthcare professionals. These medications formed an integral part of their daily routine as they navigated the demands of motherhood alongside managing their health. The background information is presented in Table 1.

Table 1. Background Information of Postpartum Mothers Living with Hypertension (N = 16)

Participant	Age (years)	Marital Status	Number of Children	Time Since Delivery	Diagnosis Period	Current Treatment	Language Used
PA	21	Married	1	3 weeks	During pregnancy	Neficard 30 mg	English & Twi
PB	24	Married	2	2 weeks	During pregnancy	Methyldopa 90 mg	Twi
PC	27	Married	3	1 month	During pregnancy	Neficard XL 30 mg	English
PD	30	Married	2	3 weeks	After delivery	Methyldopa 125 mg	English & Twi
PE	32	Married	3	2 weeks	During pregnancy	Neficard XL 90 mg	English
PF	28	Married	2	1 week	During pregnancy	Methyldopa & Neficard	Twi & Ga
PG	29	Married	2	2 weeks	During	Neficard 30	English &

					pregnancy	mg daily	Ga
PH	33	Married	4	3 weeks	During pregnancy	Neficard XL 30 mg daily	English & Twi
PI	31	Married	3	1 month	After delivery	Neficard 30 mg daily	English
PJ	35	Married	4	2 weeks	During pregnancy	Nurse-administered (unspecified)	Twi
PK	42	Married	4	2 weeks	During pregnancy	Neficard XL 90 mg	English & Twi
PL	38	Married	3	1 week	During pregnancy	Neficard XL 30 mg	English
PM	42	Married	4	1 week	During pregnancy	Neficard + Methyldopa	Twi
PN	32	Married	1	2 weeks	After pregnancy	Neficard 30 mg	English
PO	34	Married	4	1 week	After pregnancy	Neficard 90 mg	English
PP	42	Married	1	7 months	During pregnancy	Neficard XL 90 mg + Methyldopa	English

The themes and sub-themes that were generated from the data is shown in Table 2.

Table 2. Themes and sub-themes identified from the data

Main Theme	Sub-Themes	Summary Description
**1. Expectations about Living with Hypertension**	1. Optimism and hope for recovery<br>2. Fear and uncertainty about complications	Participants expressed mixed feelings about their condition. While some held optimistic beliefs about recovery through medication, healthy lifestyles, and divine intervention, others worried about future complications or recurrence in subsequent pregnancies. These expectations influenced their motivation to adhere to care.
**2. Self-Efficacy in**	1. Confidence in	Participants demonstrated varying degrees of

**Managing Hypertension**	adhering to treatment<br>2. Challenges with lifestyle modification and monitoring	confidence in managing their condition. Those with higher self-efficacy-maintained medication routines and dietary adjustments, while others struggled due to stress, limited resources, and lack of monitoring devices. Their perceived ability to manage hypertension reflected the core concept of self-efficacy within the Social Cognitive Theory, where belief in one's capacity determines persistence in health-related behavior.
**3. Environmental factors**	1. Spousal and family support<br>2. Role of healthcare providers<br>3. Social perceptions and stigma	Support from spouses, relatives, and healthcare providers played a critical role in shaping mothers' adherence and emotional well-being. Positive support enhanced motivation, whereas stigma or insensitive attitudes from the community and some healthcare workers reduced confidence and engagement in care.

The findings of this study revealed three major themes that encapsulated the multifaceted experiences of postpartum mothers living with hypertension in the Accra Metropolis. These themes, expectations, self-efficacy and environmental influences. Each theme is discussed below, supported by participants' quotations to illustrate their lived realities, and interpreted through the lens of the Social Cognitive Theory (SCT).

Expectations about living with hypertension

This theme reflects the perceptions and emotional outlook of postpartum mothers as they navigated life with hypertension following childbirth. Two sub-themes emerged: optimism and hope for recovery, and fear and uncertainty about complications. These expectations influenced how participants engaged in self-care, adhered to treatment, and adjusted psychologically. In line with Social Cognitive Theory (SCT), such expectations correspond to the construct of outcome expectancies, which guide motivation and participation in health-promoting behaviors [21, 22]. Overall, mothers' expectations reflected a combination of optimism and apprehension.

Some expressed hope and confidence in recovery, while others felt overwhelmed by the chronic nature of hypertension. According to SCT, these varying expectations influence behavior: positive outlooks encourage proactive health actions, whereas fear and uncertainty may lead to avoidance or emotional distress.

Optimism and hope for recovery

Several mothers expressed a positive perspective on recovery and effectively managing hypertension. They believed that adhering to medical advice, making lifestyle adjustments, and praying would help restore their health and allow them to fully resume maternal responsibilities. Their optimism reflects strong outcome expectations, a core SCT concept emphasizing belief in the benefits of desired behaviors. Some of the participants' quotes are below.

> *"I know if I keep taking my medicine and reduce stress, my blood pressure will come down. I am getting better, and I believe by God's grace it will be normal again."* (Participant C)
>
> *"The doctor told me it will stabilize if I follow the routine, so I don't see it as a permanent sickness. I am doing everything they say."* (Participant F)
>
> *"Sometimes I just pray and trust God that I won't live with it forever. I am hopeful."* (Participant L)

Optimism strengthened their motivation to adhere to treatment and manage stress, consistent with SCT's emphasis on expecting positive health outcomes as a driver of self-regulation.

Fear and uncertainty about complications

In contrast, some mothers reported anxiety and uncertainty about their health, describing hypertension as unpredictable and worrisome. Concerns centered on potential complications, recurrence in future pregnancies, and long-term dependence on medication. These feelings reflected low perceived control and reduced confidence, which can undermine self-efficacy and adherence to treatment. Some of the participants' quotes were:

> *"Sometimes I worry because I don't know if this BP (blood pressure) will ever go away. What if it comes back when I get pregnant again?"* (Participant A)
>
> *"When they said my pressure was high again, I got scared. I thought it meant something was seriously wrong with me."* (Participant G)
>
> *"I almost collapsed before delivery because of BP (blood pressure), so anytime I feel dizzy, I panic. I think it will happen again."* (Participant I)

These accounts illustrate how negative outcome expectations can reduce motivation and coping. Mothers who doubted their ability to prevent or control complications often experienced higher emotional distress and hesitated to implement lifestyle changes.

Self-efficacy in managing hypertension

This theme examines mothers' confidence in managing hypertension through medication adherence, lifestyle changes, and blood pressure monitoring. It reflects the self-efficacy construct of SCT, which emphasizes the belief in one's ability to perform behaviors that affect health outcomes [21, 22]. Participants' experiences ranged from strong confidence in managing their condition to struggles caused by fatigue, stress, and limited resources. Two sub-themes were identified: confidence in treatment adherence and challenges with lifestyle modification and monitoring.

Confidence in adhering to treatment

Many mothers reported confidence in following prescribed treatment routines. They consistently took medications, attended clinic appointments, and adhered to medical advice. This confidence was reinforced by information received during antenatal and post-natal care, as well as support from healthcare providers and family members. Some of the participants' quotes:

> *"I never skip my medication because I know it keeps my blood pressure down. Even when I travel, I carry it along."* (Participant D)
>
> *"The nurse taught me how to take it at the same time every day. I am used to it now; it has become part of my routine."* (Participant H)

Faith and determination strengthened some women's confidence:

> *"I tell myself I can manage it. I pray and take my drugs, and I see improvement."* (Participant J)
>
> *"When I was first diagnosed, I was scared. But now I am confident because I take the medicine and watch my diet. I know I can handle it."* (Participant N)

These accounts show that confidence grew through practice, education, and observing improvement—key aspects of self-efficacy according to SCT. Positive experiences reinforced mothers' belief in their ability to manage hypertension, fostering self-regulation and resilience.

Challenges with lifestyle modification and monitoring

Despite their commitment, many mothers found it difficult to maintain lifestyle changes such as dietary restrictions, exercise, and regular blood pressure checks. Limited finances, household

responsibilities, and lack of monitoring devices reduced their sense of control. These challenges illustrate SCT's assertion that environmental factors can limit health-promoting behaviors. Some of the participants' quotes:

> *"Sometimes I forget to check my pressure because I don't have a machine at home, and going to the hospital all the time is costly."* (Participant B)
>
> *"They said I should avoid salty food, but it's not easy because we all eat the same food at home."* (Participant F)

Some highlighted competing demands:

> *"With the baby and household chores, I hardly get time to rest or even think about what I am supposed to eat."* (Participant K)

Others noted emotional strain:

> *"At times I feel tired and stressed, and that alone makes my pressure rise. I try to follow the advice, but it's not always possible."* (Participant M)

Postpartum mothers showed varying levels of confidence in managing hypertension. Those with strong self-efficacy consistently adhered to medications and engaged in positive health behaviors, whereas others struggled with lifestyle modifications due to stress, limited resources, and inadequate support. Consistent with SCT, self-efficacy is essential for effective hypertension management as it influences motivation, persistence, and adaptive coping.

Environmental factors

This theme examines how external environmental factors, including spousal involvement, family support, relationships with healthcare providers, and social perceptions, shaped mothers' hypertension management. According to Social Cognitive Theory (SCT), health behaviors result from continuous interactions between personal factors, environmental conditions, and behavioral responses [21, 22]. In this study, environmental influences acted as both enabling and constraining factors, affecting adherence, emotional well-being, and motivation. Environmental factors significantly shaped mothers' management of postpartum hypertension. Spousal and family support motivated adherence, and positive healthcare interactions reinforce self-efficacy. Conversely, stigma and negative social perceptions create emotional distress and secrecy. SCT underscores that behavior change and self-management are influenced by both social and environmental contexts.

Three sub-themes emerged: spousal and family support, role of healthcare providers and social perceptions and stigma.

Spousal and family support

Many mothers acknowledged that support from spouses and family was essential to managing hypertension effectively. Encouragement, reminders to take medication, financial help, and emotional reassurance were critical for adherence and recovery. Supportive family relationships enhanced confidence and reduced stress, reinforcing positive health behaviors. Some of the participants' quotes were:

> *"My husband reminds me to take my medicine and even buys them for me when I forget."* (Participant F)
>
> *"My mother helps me with the baby so I can rest and take my medication. Without her, I would have broken down."* (Participant J)
>
> *"When my pressure was high, my husband was always by me, encouraging me and saying I would be fine."* (Participant C)

However, some mothers reported limited or absent support. One shared.

"I live alone with my child; there is no one to check on me or remind me to take my drugs." (Participant R)

These accounts illustrate SCT's principle of reciprocal determinism, showing how supportive social environments enhance self-efficacy and health-promoting behaviors, while the lack of support can increase vulnerability and reduce motivation.

Role of Healthcare Providers

Healthcare professionals, particularly nurses and midwives, played a central role in mothers' recovery. Participants valued guidance, emotional reassurance, and professional care during clinic visits. Positive interactions reinforced confidence in self-care and strengthened adherence, consistent with SCT's emphasis on modelling and reinforcement. Some of the participants' quotes were.

> *"The nurses are patient with me. They check my blood pressure and explain what I should do to bring it down."* (Participant A)
>
> *"Sometimes I go there feeling weak, and they comfort me. They even share stories of others who are doing well to encourage me."* (Participant K)
>
> *"The midwife calls me when I miss my appointment and advises me not to stop my medication. That has helped me a lot."* (Participant S)

Some mothers, however, experienced limited attention due to staff workload:

> *"Sometimes the nurses are too busy. They just check the pressure and give the drugs without much talk."* (Participant M)

These narratives highlight that consistent, empathetic healthcare interactions can strengthen self-efficacy, whereas insufficient support may undermine trust and engagement.

Social perceptions and stigma

Some mothers reported encountering negative social judgments and stigma regarding their hypertension. Community members and even healthcare staff sometimes viewed them as "too young" to have high blood pressure, causing feelings of shame and isolation and discouraging openness about their condition. Below are some of the quotes from the participants.

> *"When I went to the pharmacy, they said, 'Ah, small girl like you with blood pressure?' It made me feel bad."* (Participant P)
>
> *"Some people think only old people get high blood pressure, so when they see me taking medicine, they laugh."* (Participant D)
>
> *"I hide my drugs when friends visit because I don't want questions."* (Participant H)

These accounts show how stigma and misconceptions can disrupt self-regulation and reduce motivation. SCT highlights that negative environmental cues may hinder health-promoting behaviors, suggesting that public education could help normalize postpartum hypertension.

Discussion

This study explored postpartum mothers' expectations regarding the management of hypertension. The findings revealed that the participants had diverse expectations of their diagnosis. Some were optimistic, expressing confidence on medication and lifestyle modifications as well as faith in divine interventions leading to recovery. Others experienced fear, anxiety, and uncertainty about complications or recurrence in future pregnancies. These expectations shaped their emotional well-being, motivation, and adherence to treatment. According to Social Cognitive Theory (SCT), outcome expectations as well as beliefs about the likely results of one's actions play a critical role in health behaviors [20-22, 28]. The study found that mothers who believed that consistent medication use, regular monitoring, and stress management would improve their health demonstrated greater commitment to self-care. Conversely, those who doubted the effectiveness of these measures or feared long-term complications showed lower confidence and reduced adherence. This finding is consistent with the assertions of previous studies [23-25] that stated that positive expectations foster persistence in managing chronic health conditions. These findings also align with prior research. For example, [26] reported that Ghanaian women with hypertensive disorders in

pregnancy who anticipated positive outcomes were more likely to comply with postpartum care and medication. Similarly, Mensah and Obeng [19] found that women who believed their blood pressure could normalize after childbirth were more motivated to follow medical advice and adopt healthy behaviors. In contrast, fear of complications negatively influenced engagement in care, consistent with [11, 26], who noted that women perceiving hypertension as a lifelong condition often expressed frustration and hopelessness and thus reducing adherence.

Spirituality also played a significant role in shaping expectations. Many participants relied on prayer and faith in God, interpreting their recovery as partly dependent on divine intervention. This supports [27-33] observation that Ghanaian mothers often integrate spirituality into health-seeking behaviors, using faith as a source of emotional strength. Within the SCT, spiritual beliefs can serve as self-reinforcement, sustaining motivation and coping [33]. However, the coexistence of hope and fear illustrates the psychological complexity of adjusting to postpartum hypertension. Participants who were uncertain about recurrence or long-term effects tended to exhibit lower confidence in managing their condition, echoing findings by [28-30], who noted that prior experiences with pre-eclampsia heightened anxiety and reduced adherence to follow-up care. From a SCT perspective, low outcome expectations can weaken self-efficacy and hinder proactive health behaviors [21-22].

Furthermore, the results from this study showed that the participants with higher self-efficacy described maintaining medication schedules, attending clinic appointments, and implementing dietary recommendations. Positive health outcomes, ongoing guidance from healthcare providers, and family encouragement reinforced their confidence, supporting [18, 29] claim that mastery experiences and social reinforcement enhance self-regulation in health management. However, some of the participants reportedly struggled to sustain dietary restrictions, regular exercise, and blood pressure monitoring due to limited financial resources, caregiving responsibilities, and psychological fatigue [2, 4, 27]. These barriers align with [29-32] who reported that economic hardship and family obligations hindered postpartum women's self-care in Ghana. Similarly, Obeng et al. [15] found that low self-efficacy among women with chronic conditions was associated with inconsistent medication use and delayed health-seeking behavior.

In addition, supportive family environments were found to foster motivation and confidence, while isolation and stress undermined self-management similar to those stated in other studies [14, 29-31]. Emotional resilience and faith were additional factors identified by the

participants. Some of the mothers reported that prayer and positive thinking helped them persevere with treatment despite challenges. This spiritual motivation aligns with Yeboah et al. [18], who observed that faith-based coping reinforces self-efficacy by promoting hope and determination. Overall, self-efficacy was dynamic, evolving through experience, social reinforcement, and health outcomes. Confidence increased with observable health improvements or affirmation from healthcare providers and family, while unmet expectations, stress, or lack of support diminished motivation. These findings echo Mensah et al.'s work [19], who highlighted that self-efficacy develops through continuous learning and reinforcement in the social context.

The study found that mothers who received encouragement, reminders, and practical assistance from spouses and family members were more likely to adhere to medications and maintain healthy routines. This aligns with [18, 29-31], who observed that spousal and family support fosters self-efficacy, emotional well-being, and adherence among postpartum women managing chronic illnesses in Ghana. Evidence from Ghana also shows that access to supportive environments and self-monitoring tools improves mothers' confidence in managing hypertension [6, 26, 28]. Conversely, mothers lacking family or partner support reported isolation, emotional distress, and difficulty following medical advice, consistent with other studies [29, 30], who noted that limited social support contributes to poor self-management and heightened anxiety. Similarly, healthcare providers who provided empathetic communication, education, and follow-up increased the participants' confidence and trust in treatment. Positive interactions reinforced adherence by providing guidance and reassurance, echoing Appiah et al. [20] work, who emphasized that supportive midwife-patient relationships enhanced treatment adherence and emotional resilience. Conversely, some participants reported rushed consultations and limited attention from overburdened staff, reducing satisfaction and motivation, reflecting health system constraints as highlighted by [34].

The study showed that social stigma and misconceptions about hypertension further influenced experiences of the participants. Several mothers reported ridicule or misunderstanding from peers who perceived hypertension as a disease of older adults. They claimed that the stigmatization led to secrecy, shame, and reluctance to seek help, supporting Boateng and Nartey [27], who noted that chronic illness stigma fosters emotional distress and avoidance behaviors. SCT suggests that such negative social cues disrupt self-regulation and reduce engagement in health-promoting behaviors. Overall, the findings indicate that the environment

profoundly affects postpartum mothers' management of hypertension. Supportive relationships with family, spouses, peers, and healthcare providers fostered motivation, emotional stability, and adherence, while social stigma and healthcare system limitations undermined confidence and self-care. SCT underscores that health behaviors are shaped through interactions between individuals and their social environments. Strengthening supportive ecosystems through family education, community awareness, and improved healthcare engagement is crucial. Health policies should emphasize patient-centered care, spousal involvement, and stigma reduction to enhance self-efficacy, adherence, and overall well-being among postpartum women with hypertension.

Limitations of the study

The study design was crosse-sectional, and the findings only describe the experiences and do not infer causation. More robust techniques such as longitudinal designs are encouraged in subsequent studies.

The study was hospital-based and did not involve other women who were not receiving care at the study settings. The findings cannot represent the views of all women diagnosed with hypertension during the postnatal period.

The data could have been affected by recall bias even though efforts were made to ensure the participants gave a vivid account during the study

Furthermore, the findings like most questionnaire-based studies could be affected by social desirability issues. However, the researchers asked the participants to give a vivid account of their experiences as per the interview guide and this reduced the social desirability issues.

Conclusion and recommendations

Effective management of postpartum hypertension requires patient-centered and holistic interventions that enhance women's self-efficacy, strengthen family and community support, reduces stigma and improve midwife–client communication.

It is recommended that the Ghana Health Service design targeted postpartum hypertension education programs aimed at reducing social stigma and integrates the role of spouses and social networks of clients including faith-based organizations to improve compliance with treatment and lifestyle modifications.

Abbreviations

GARH-Greater Accra Regional Hospital

SCT- Social Cognitive Theory

References

1. World Health Organization. (2020). *Hypertensive disorders of pregnancy: Key facts and recommendations*. https://www.who.int
2. World Health Organization. (2021). *Trends in maternal mortality: 2000–2020*. https://www.who.int/publications
3. Amadu, A. B., Konlan, K. D., Amadu, J. B., & Dzansi, G. (2025). Treatment Adherence and Quality of Life of Adults Living With Hypertension in Rural Ghana. *Nursing open*, *12*(3), e70198. https://doi.org/10.1002/nop2.70198
4. Konlan, K. D., Konlan, K. D., Abdulai, J. A., Saah, J. A., Doat, A. R., Amoah, R. M., & Mohammed, I. (2023). The relationship between trust, belief and adherence among patients who complain of hypertension in Ghana. *Nursing open*, *10*(9), 6205–6214. https://doi.org/10.1002/nop2.1855
5. Konlan K. D. (2025). Burnout is a predictor of high ten-year risk of cardiovascular disease among health workers in Ghana. *BMC public health*, *25*(1), 2748. https://doi.org/10.1186/s12889-025-23834-6
6. Konlan, K. D., Afam-Adjei, C. J., Afam-Adjei, C., Oware, J., Appiah, T. A., Konlan, K. D., & Bella-Fiamawle, J. (2020). Practice and Sociodemographic Factors Influencing Self-Monitoring of Blood Pressure in Ghanaians with Hypertension. *International journal of chronic diseases*, *2020*, 6016581. https://doi.org/10.1155/2020/6016581
7. Konlan, K. D., Baku, E. K., Japiong, M., & Amoah, R. M. (2020). Maternal knowledge and attitudes toward pregnancy-induced hypertension in Ghana. *PLOS ONE, 15*(8), e0237385. https://doi.org/10.1371/journal.pone.0237385
8. Konlan, K. D., Konlan, K. D., Abdulai, J. A., Saah, J. A., Doat, A. R., Amoah, R. M., & Mohammed, I. (2023). The relationship between trust, belief and adherence among patients who complain of hypertension in Ghana. *Nursing open*, *10*(9), 6205–6214. https://doi.org/10.1002/nop2.1855
9. Lartey, S., Owusu, P., & Aboagye, P. (2023). Social determinants influencing hypertension control in postpartum mothers. *Journal of Public Health in Africa, 14*(2), 233–241. https://doi.org/10.4081/jphia.2023.2312
10. Smith, A. L., Johnson, T., & Adelman, J. (2022). Postpartum hypertension: A contemporary review of management and outcomes. *American Journal of Obstetrics & Gynecology, 227*(2), B2– B10. https://doi.org/10.1016/j.ajog.2022.04.011

11. Adu-Bonsaffoh, K., Seffah, J. D., & Obed, S. A. (2023). Maternal outcomes of hypertensive disorders in pregnancy in Ghana: A tertiary hospital review. *PLOS ONE, 18*(2), e0281123. https://doi.org/10.1371/journal.pone.0281123
12. Hammond, A., Owusu, A., & Tuffour, E. (2021). Maternal understanding of postpartum hypertension and its complications in urban Ghana. *BMC Women's Health, 21*, 355. https://doi.org/10.1186/s12905-021-01463-2
13. Razak, S., Mohammed, I., & Fuseini, A. (2023). Cultural influences on maternal help-seeking after hypertensive disorders of pregnancy. *Women & Health, 63*(5), 452–465. https://doi.org/10.1080/03630242.2023.2205417
14. Kasonde, J., Mweemba, P., & Sakala, B. (2023). Women's lived experiences of managing postpartum hypertension in sub-Saharan Africa: A qualitative synthesis. *International Journal of Gynecology & Obstetrics, 162*(1), 101–110. https://doi.org/10.1002/ijgo.14567
15. Obeng, P., & Agyapong, S. (2021). Health system barriers to maternal follow-up after hypertensive disorders of pregnancy. *Health Services Research and Managerial Epidemiology, 8,* 1–10. https://doi.org/10.1177/23333928211032412
16. Premkumar, A., Chandraharan, E., & Khalil, A. (2020). Hypertension in pregnancy: Best practices in postpartum follow-up. *Obstetrics & Gynecology Clinics, 47*(4), 669–683. https://doi.org/10.1016/j.ogc.2020.08.006
17. Boateng, P., & Nartey, E. (2023). Peer support groups and resilience among women with chronic conditions in Ghana. *Ghana Medical Journal, 57*(2), 101–110. https://doi.org/10.4314/gmj.v57i2.6
18. Yeboah, P., Ofori, M., & Ansah, E. (2024). Self-efficacy and treatment adherence among postpartum women with hypertension in Ghana. *BMC Women's Health, 24*, 118. https://doi.org/10.1186/s12905-024-03240-y
19. Mensah, R., Osei, A., & Turkson, J. (2023). Experiences of postpartum women with hypertensive disorders: A phenomenological inquiry. *BMC Pregnancy and Childbirth, 23,* 602. https://doi.org/10.1186/s12884-023-05845-6
20. Appiah, K., Mensah, S., & Tetteh, R. (2022). Midwife–patient interactions and adherence to postpartum care in Ghana. *International Journal of Nursing Practice, 28*(4), e13012. https://doi.org/10.1111/ijn.13012
21. Bandura, A. (2020). Social cognitive theory: An agentic perspective. *Annual Review of Psychology, 71,* 1–26. https://doi.org/10.1146/annurev-psych-010419-050833

22. Bandura, A. (2023). Advances in self-efficacy theory. *Journal of Applied Psychology, 108*(4), 621–639. https://doi.org/10.1037/apl0001139
23. Salisu, R. A., & Ansu-Kyeremeh, K. (2021). The role of family support in postpartum women's adjustment to chronic illness. *BMC Women's Health, 21*, 421. https://doi.org/10.1186/s12905-021-01539-z
24. Hammond, A., Owusu, A., & Tuffour, E. (2021). Maternal understanding of postpartum hypertension and its complications in urban Ghana. *BMC Women's Health, 21*, 355. https://doi.org/10.1186/s12905-021-01463-2
25. Brani, P., Mrvoljak-Theodoropoulou, I., Pechlivani, F., Gourounti, K., Iliadou, M., Palaska, E., Antsaklis, P., Drakakis, P., & Dagla, M. (2024). Mothers' Breastfeeding Self-Efficacy after a High-Risk or Normal Pregnancy: A Greek Longitudinal Cohort Study. *European journal of investigation in health, psychology and education*, *14*(6), 1803–1820. https://doi.org/10.3390/ejihpe14060119
26. Addo, P., Owusu, A., & Boateng, J. (2022). Postpartum care adherence among Ghanaian women with hypertensive disorders: The role of outcome expectations. *Ghana Medical Journal, 56*(3), 145–153. https://doi.org/10.4314/gmj.v56i3.5
27. Boateng, P., & Nartey, E. (2023). Peer support groups and resilience among women with chronic conditions in Ghana. *Ghana Medical Journal, 57*(2), 101–110. https://doi.org/10.4314/gmj.v57i2.6
28. Saaka, M., Mohammed, A., & Issah, A. (2020). Patterns of postpartum care utilization among women with pregnancy-related complications in northern Ghana. *PLOS ONE, 15*(10), e0240659. https://doi.org/10.1371/journal.pone.0240659
29. Tandoh, M., Arhin, V., & Amu, H. (2021). Coping mechanisms among postpartum women experiencing chronic health challenges in Ghana. *Midwifery, 102*, 103123. https://doi.org/10.1016/j.midw.2021.103123
30. Teye, E., Obeng, P., & Agyekum, S. (2023). A qualitative exploration of barriers to follow-up among mothers with hypertensive disorders of pregnancy. *BMC Pregnancy and Childbirth, 23*, 711. https://doi.org/10.1186/s12884-023-05942-6
31. Nyarko, K., Tenkorang, E., & Adwoa, S. (2023). Social support and self-efficacy among women managing postpartum hypertension. *African Journal of Reproductive Health, 27*(3), 88–99. https://doi.org/10.29063/ajrh2023/v27i3.8
32. Mwinbam, B., Abasimi, E., & Der, R. (2021). Spiritual coping and health behavior among Ghanaian women with chronic diseases. *BMC Women's Health, 21,* 334. https://doi.org/10.1186/s12905-021-01438-3

33. Lamptey, P., Obiri-Yeboah, D., & Addo, J. (2021). Psychological distress and coping strategies among postpartum women with chronic health conditions. *Ghana Medical Journal, 55*(4), 210–219. https://doi.org/10.4314/gmj.v55i4.7
34. Antwi, F. A., & Appiah, S. (2020). Experiences of disrespectful maternity care among women in Ghana: A qualitative inquiry. *BMC Pregnancy and Childbirth, 20*, 657. https://doi.org/10.1186/s12884-020-03351-0

Supporting information

1. S2 File: Interview guide

Click here to access/download
Supporting Information
S2.docx
