# Supplementary material for: Lived experiences of postpartum hypertensive women in Accra, Ghana
Source: PLoS One. 2026 May 27;21(5):e0349781. doi: 10.1371/journal.pone.0349781 (PMC13215485; doi:10.1371/journal.pone.0349781)
Supplement: Attachment — Submitted filename: Reviewer Report 8.12.25.pdf [file pone.0349781.s003.pdf]

Reviewer Report – PLOS ONE

Manuscript Title: Lived experiences of postpartum women diagnosed with hypertension receiving care in a resource-constrained setting in Accra, Ghana

Manuscript Number: PONE-D-25-62035

Summary of the manuscript

This manuscript investigates the lived experiences of postpartum women diagnosed with hypertension receiving care in a resource-constrained setting in Accra, Ghana, using a qualitative descriptive phenomenological design based on the social cognitive theory. The author collected data from 16 women using semi-structured in-depth interviews. The manuscript content demonstrates scientific rigor for a qualitative study.

Background

- The current background emphasizes the epidemiology and burden of postpartum hypertension but does not sufficiently address the lived experiences of affected mothers.
- Strengthening the background to reflect existing evidence on women's experiences and clearly identifying the gap your study intends to fill will enhance the rationale.
- The theoretical framework is not described before its application in the study. Describe the constructs and how they will be applied to inform the thematic analysis.

Methods

- The study design is appropriate, and the sampling is well described, including how data saturation was achieved.
- The study design description appears incomplete.
- In the inclusion criteria, describe how you will assess to confirm a cognitively stable mother
- The authors have not included the NVivo data codes or codebook used in the analysis. Without this information, the transparency of the analytic process is limited, and it is unclear which themes were most recurrent or how the relative weight of each theme was determined.

Results

- The results are well captured with participants' demographics clearly displayed in a table, and participants' excerpts are also indicated as appropriate for each subtheme/theme.
- A participant who was 7 months postpartum appears inconsistent with the rest of the sample, as the other mothers were 1 week to 1 month postpartum. Can this participant be considered postpartum at this time?

Discussion and Conclusions

- The comparison with other studies is noted, but the author's independent scholarly contribution is not clear. The discussion should more explicitly articulate the significance of the findings and provide a clear response to the "so what" question.
- In the discussion, the study design changes to cross-sectional instead of descriptive phenomenology

- The discussion mentions that the study explored the expectations of the postpartum mothers instead of the experiences.
- The limitations are well articulated.
- The conclusion may need strengthening. Conclude based on the study findings

Ethical Considerations: The ethical consideration details are well captured and complete.
