# Supplementary material for: Lived experiences of postpartum hypertensive women in Accra, Ghana
Source: PLoS One. 2026 May 27;21(5):e0349781. doi: 10.1371/journal.pone.0349781 (PMC13215485; doi:10.1371/journal.pone.0349781)
Supplement: Attachment — Submitted filename: Response_to_reviewers__auresp_2.pdf [file pone.0349781.s006.pdf]

University of Ghana
College of Health Sciences
School of Nursing and Midwifery
Department of Adult Health

26th March, 2026

The Editor
PLOS ONE

Dear Sir/Madam,

Response to review

General comments of authors

We have addressed all the comments of the reviewers as suggested and we hope the revised manuscript meets the standards for publications.

Reviewer #1:

Reviewer #1: Reviewer comments

The authors have adequately addressed all the concerns raised in my initial review. The revisions have strengthened the manuscript, particularly in terms of clarity and methodological description. I have no major concerns and support the publication of the manuscript, subject to the following minor revisions:

Minor edits:

1. The abstract exceeds the recommended word limit (approximately 362 words). Please revise the conclusion and recommendations to be more concise.

2. Please clearly specify the source of Figure 1 (the theoretical framework), indicating whether it was developed by the authors, adapted, or adopted from existing literature, and provide an appropriate reference where applicable.

<u>Authors' response to reviewer 1</u>

We are grateful for the comments of the reviewer and have addressed all the comments in the revised manuscript as specifically indicated below

Minor edits:

1. We have worked on the abstract on page 2 of the revised manuscript to make same concise as recommended by the reviewer

2. We have specified that Figure 1 (the theoretical framework) was adopted and provided the appropriate citation. This is found on page 6 of the revised manuscript.

Reviewer #2:

The authors have attended to all recommendations and the article is up to standard. I recommend acceptance of the manuscript.

<u>Authors' response to reviewer 2</u>

We are grateful for the reviewer's comment of recommending acceptance of the manuscript and we have addressed the minor comments of the other reviewer.

We hope the revised manuscript will meet the standards for publication.

Thank you

Yours sincerely,
Dr. Kennedy Dodam Konlan
(Corresponding author)
