# Supplementary material for: Lived experiences of postpartum hypertensive women in Accra, Ghana
Source: PLoS One. 2026 May 27;21(5):e0349781. doi: 10.1371/journal.pone.0349781 (PMC13215485; doi:10.1371/journal.pone.0349781)
Supplement: Attachment — Submitted filename: Reviewer report 8.4.26.pdf [file pone.0349781.s007.pdf]

Reviewer Report – PLOS ONE

Manuscript Title: Lived experiences of postpartum women diagnosed with hypertension receiving care in a resource-constrained setting in Accra, Ghana

Manuscript Number: PONE-D-25-62035

Dear Editor,

Thank you for the opportunity to review the manuscript titled *"Lived Experiences of Postpartum Hypertensive Women."*

I have thoroughly reviewed the revised manuscript and the authors' responses to the reviewers' comments. I am confident that the authors have sufficiently addressed all the concerns raised in the previous review.

In its current form, the manuscript meets the standards for publication. I therefore recommend that it be accepted for publication in PLOS ONE.

Thank you once again for the opportunity to contribute to the peer-review process.

Kind regards,
Dr. Serah Wachira
Senior Instructor
Aga Khan University School of Nursing and Midwifery
