# Supplementary material for: Lived experiences of postpartum hypertensive women in Accra, Ghana
Source: PLoS One. 2026 May 27;21(5):e0349781. doi: 10.1371/journal.pone.0349781 (PMC13215485; doi:10.1371/journal.pone.0349781)
Supplement: Attachment — Submitted filename: Reviewer Report 28.1.26.docx [file pone.0349781.s005.docx]

**Reviewer Report – PLOS ONE**

**Manuscript Title**: Lived experiences of postpartum women diagnosed with hypertension receiving care in a resource-constrained setting in Accra, Ghana

**Manuscript Number**: PONE-D-25-62035

**Reviewer comments**

The authors have adequately addressed all the concerns raised in my initial review. The revisions have strengthened the manuscript, particularly in terms of clarity and methodological description. I have no major concerns and support the publication of the manuscript, subject to the following minor revisions:

**Minor edits:**

1. The abstract exceeds the recommended word limit (approximately 362 words). Please revise the conclusion and recommendations to be more concise.
2. Please clearly specify the source of Figure 1 (the theoretical framework), indicating whether it was developed by the authors, adapted, or adopted from existing literature, and provide an appropriate reference where applicable.
